# “Brought to life through imagery” – animated graphic novels to promote empathic, patient-centred care in postgraduate medical learners

**DOI:** 10.1186/s12909-021-02491-4

**Published:** 2021-01-21

**Authors:** Travis Sutherland, Dorothy Choi, Catherine Yu

**Affiliations:** 1 University of Toronto, Toronto, ON M5S 1A8 Canada; 2 Li Ka Shing Knowledge Institute of St. Michael’s Hospital, Toronto, ON M5B 1W8 Canada; 3 Unity Health Toronto, Toronto, ON M5B 1W8 Canada; 4 Division of Endocrinology & Metabolism, St. Michael’s Hospital, 30 Bond Street, Toronto, ON M5W 1B8 Canada; 5 Faculty of Medicine and Dalla Lana School of Public Health, University of Toronto, 30 Bond Street, Toronto, ON M5W 1B8 Canada; 6 Li Ka Shing Knowledge Institute of St. Michael’s Hospital School of Public Health, University of Toronto, 30 Bond Street, Toronto, ON M5W 1B8 Canada

**Keywords:** Post-graduate medical education, Comics, Graphic novels, Empathy training, Communications training, Burnout

## Abstract

**Background:**

Empathy levels decline through medical training. This has been associated with poor patient and physician outcomes, and strategies to combat this decline are increasingly recognized as critical aspects of medical education. The aim of this study was to qualitatively determine factors associated with empathy decline, and to assess the impact of a comics/graphic novel-based curriculum on enhancing empathy and a patient-centered approach to care in post-graduate medical learners.

**Methods:**

Fourth and fifth year residents in the Adult and Pediatric Endocrinology and Metabolism Program at the University of Toronto were recruited from the 2017 cohort of the Empathy, Humanism & Comics course. Participants completed a 12-month curriculum, viewing a total of four animated graphic novels over six sessions. At the end of the course participants were interviewed either individually or in a focus group. A coding framework of emerging themes was developed based on consensus between the three authors using a qualitative descriptive approach and the constant-comparison method.

**Results:**

Analysis of coded interview data revealed four themes. 1. The curriculum accurately reflected and addressed issues in real world medical practice; 2. The comics curriculum facilitated holistic development; 3. Participants appreciated the comics as an educational medium; 4. Participant feedback on the curriculum. The importance of empathy was noted, while participants acknowledged their own empathy decline and increased burnout. Stressors included increasing responsibility, long work hours, and competing work-life responsibilities. They felt the sessions developed resilience, an appreciation for the patient perspective, and communication skills. They appreciated the comics as a novel and engaging educational modality. Feedback on the effectiveness and relevancy of the curriculum was variable.

**Conclusions:**

Residents appreciated sharing difficult experiences and seeking support. They acknowledged the curriculum as a commitment to wellness and felt it reduced burnout and improved empathy. The comics were viewed as an effective reminder of the patient perspective. Variable curriculum feedback highlights the challenge in designing a course for adult learners. Future investigations may include the development and incorporation of similar curricula in other post-graduate residency training programs.

## Background

The importance of empathy in the physician-patient relationship has long been recognized [1]. Empathic care has been linked to increased patient satisfaction and improved adherence to therapy [2, 3], improved clinical outcomes [2, 4], and lower malpractice liability [2]. Given these benefits, many organizations in medical education, including the Association of American Medical Colleges and the American Board of Internal Medicine, recommend a focus on empathy in medical education [5, 6]. However, empathy among medical students declines as they progress through training [6, 7]. This decline continues into post-graduate education, significantly impacting the mental wellness and clinical performance of residents [7]. The etiology is thought to be multifactorial, including loss of idealism, heavy workload, poor social support, and conflicting professional-personal responsibilities [7, 8]. Alongside reduced empathic practice, studies reporting resident burnout and depression abound [8]. To address these concerns, strategies to teach and maintain empathy have been developed [2]. Several interventions, including the use of patient narratives and creative arts have been effective in improving empathy in undergraduate medical students [2]. The long-term benefits of these studies are unclear however, and follow up studies in post-graduate medical learners have not been conducted.

Graphic stories, or comics, are a novel educational modality [9]. They combine text with imagery to convey immediate and evocative understanding of the material. Our team previously developed online animated comics depicting a patient’s experience living with type 2 diabetes. We previously investigated the impact of this intervention on undergraduate medical students’ approach to patient interactions [10, 11]. Pre-clerks found the comics to be a useful reminder of empathy [10]. Clerkship students identified the value of empathy in the patient-physician relationship, giving it equal value to physician competence and medical knowledge [11]. Of note, most of these students acknowledged the difficulty in maintaining empathy in all clinical scenarios and many anticipated a decline in empathy as their training progressed.

The utilization of graphic stories as a tool for empathy education in postgraduate medical learners has not been well established. The primary aim of our study was to qualitatively assess factors affecting empathy in postgraduate training and investigate the impact of educational animated comics on affecting empathy changes and learning processes.

## Methods

This qualitative study aimed to answer the research questions: What factors contribute to burnout in a cohort of fourth- and fifth-year endocrinology residents, and would an empathy training curriculum delivered with graphic novels affect empathy-based practice and burnout? Fourth- and fifth-year residents of the Adult and Paediatric Endocrinology and Metabolism Residency Program at the University of Toronto who had attended the 2017–2018 6-part comics curriculum were eligible to participate. Recruitment was done by either the principal researcher or research coordinator via a recruitment email. It was estimated that 5–10 participants would be necessary to achieve saturation. Ethics approval from the Research Ethics Board at the University of Toronto was obtained prior to initiating the study.

The program consisted of six modules. In each module, students read a case vignette and reflected through a written exercise. Afterwards, they were presented with a comic depicting a patient’s perspective of living with a chronic disease and were guided in small groups through key concepts of empathy, patient-centred care, shared decision-making, communication, burnout and resilience. Our team created four animated comics with learning objectives based on literature review on diabetes management [12, 13], which served as a foundation for specific discussion questions. Residents were provided with post-session exercises/reflections to solidify concepts.

The first session focused on burnout with a comic entitled “Daily Grind”, showing the burnout of a patient trying to adhere to lifestyle recommendations [14]. Residents discussed the importance of physician empathy on patient care. Post-session, students were asked to reflect on their own burnout and to consider alternative ways to approach challenging situations. The second session covered barriers to change with the comic “Not the Needle” [15], focused on a patient’s fear around starting insulin. Residents discussed how physicians can act as communicators and health advocates to enable behavioural change. Residents were tasked with learning a patient’s story and identifying barriers to behavioural change. The third session focused on shared decision making with a comic entitled “So many things change …” , showing a patient and health care provider deciding on lifestyle changes for diabetes management. The discussion focused on how shared decision making can facilitate success in behaviour change. Residents reflected on how to incorporate shared decision making into their own practices. The fourth session focused on physician compassion with the first part of a comic entitled “What do you mean I have diabetes?”, showing a patient receiving a diagnosis of diabetes. Discussion focused on the difficulty of breaking bad news, and residents reflected on their own clinical experiences. The fifth session revisited burnout with the conclusion of “What do you mean I have diabetes?”. Participants discussed how the leader role can be used to manage burnout. Students were asked to re-assess their own burnout at the conclusion of the course, burnout they’ve witnessed in others, and their own strategies to manage burnout. The final session focused on resilience, with strategies to promote resilience in their patients and themselves.

This was a qualitative study using focus group and individual interview methodology.

Specific outcomes measured included how the curriculum impacted residents as physicians, as teachers, and as people; which aspects of the curriculum participants found must valuable; and how residents felt the curriculum affected their levels of empathy and approach to patients. General feedback was also obtained.

Our primary source of data included a year-end evaluation (after 12 months), which was conducted via focus groups and personal interviews as prospective data. Data collection took place over a three-month period following course completion and was performed by research assistants with no prior association with participants. The primary author (C. Yu) was not present during data collection and did not participate in focus group/interview transcription to protect the confidentiality of the participants. Semi-structured interview guides (Appendix 1) were used to explore participant’s perspectives, application of the material, empathy, and impact on learner outcomes. Transcripts from the focus group and interviews were coded and analyzed. The semi-structured interview guides underwent iterative refinement as new themes emerged from transcript coding.

Interview transcripts and online written responses were coded between three independent reviewers (TS, CY, and DC) following an inductive thematic approach [16]. A coding framework of emerging themes was developed based on author consensus, using a qualitative descriptive approach and the constant-comparison method (Appendix 2) [17, 18].

## Results

A total of six PGY4/5 students from the Adult and Paediatric Endocrinology and Metabolism program participated in the study. We identified four major subthemes as follows (representative quotes in Table 1):

**Table 1:** Themes and representative quotes

Theme/subtheme	Representative quote(s)
**1. The curriculum accurately reflected and addressed issues in real world medical practice**	
1a. Level of empathy	“I think that I am reflective of the general literature that empathy does kind of decline throughout medical education … I think that there’s a little bit of cycle, so times of higher stress and higher burnout, are the times when empathy becomes the hardest but then you kind of get back on your feet again.” – PPI8
1b. Other humanities/communications training	“I think it was a component of the medical school curriculum … it was kind of a longitudinal part of our non-clinical course. So that was kind of the only formal training I’ve had in empathy.” – PPI6
**2. The comics curriculum facilitated holistic development**	
2a. Demonstration of institutional commitment to wellness	“I think it is valuable to have these sessions to show that like y’know it’s still supported like, she’s done this research and it’s been..y’know funded by grants so like people still care about that, and that to have the time and space to have refreshments and to spend that time talking about this is valuable.” – FG1
2b. Provided techniques to practice patient centered approach	“I think it probably impacted me the most as a physician. It just made me more cognizant of specific interactions and strategies with patients. I wouldn’t say it’s something I thought about all the time but particularly when there was a challenging case or a patient who I felt I didn’t have a good connection with for whatever reason.” – FG2
2c. Fostered resilience	“I think the most important part was normalization of experiences and the chance to talk about it with the other students, having that safe space and time to do that. And I find that just talking about it itself could be somewhat therapeutic” – PPI8
2d. Provided tools to help students tackle challenging situations	“It also gave me more tools to be able to talk to them about ok, this patient isn’t following recommendations or wants to leave against medical advice, or is feeling burnt out. You need more tools about how to talk to students about different ways of approaching those situations.” – PPI8
**3. Participants appreciated the comics as an educational medium**	
3a. Positive aspects of comics	“Part of what was engaging about the comic was the fact that it was a bit of a unique form of art, and in turn was helpful to pay attention” – PPI6 “Yeah I felt the comics were really good seeing both sides, and seeing what it’s like from the patient side to live with diabetes...” – PPI8
3b. As an educational medium	“… you can say a lot in a picture which could take a chapter of a textbook and get that message across really quickly. And I think that’s the real power of it really.” – PPI7
3c. Refinement of material	“Like a framework to think of how to reach those goals, so in that way I dunno if that’s something that could be added to this … so it’s a bit more technical and to show us how to reach those goals cause I think we all want to be compassionate, it’s just how do you do it is the issue” – FG1 “The part where they’re showing you sort of what not to do and what to do sometimes it’s a bit too obvious or maybe at too much of a basic level … so maybe if that could delve more into some of the subtleties … not as like one disastrous situation and one amazing situation, but something more subtle where the lines are more blurred might be a bit more thought provoking I think” – FG3
**4. Participant feedback on the curriculum**	
4a. Value of reflective exercises and discussion	“I liked the reflective exercises cause then you really think about your own patients and your own interactions … I can still think of the patients I discussed earlier in the year I think that was probably the most beneficial for me personally” – FG2
4b. Relevance of curriculum	“I felt the messages that they were delivering were relatively fundamental. I’d like to think that most of the people watching the comics and discussing them already had a lot of those clinical skills. I appreciated medical school, but then you sort of forget it as a clinician and the comics probably brought people up to a level” – PPI8 “they do focus on some challenges that might be specific to patients with a chronic illness like diabetes. So in that way … it’s more like directly applicable to our future careers” – FG2
4c. Facilitator of curriculum integration	“I think the timing of them in the academic half day works really well, cause this is the only time really in the week that we have protected time for these kinds of things.” – FG3 “It didn’t feel didactic, I mean the way the sessions were delivered was more of a discussion piece where you could also bring your own ideas and experiences to it. So in that sense it felt more of an informal formal program, if that makes sense” – PPI7

### The curriculum accurately reflected and addressed issues in real world medical practice

Participants were asked to describe their perspective on empathy in residency and beyond, as well as to outline previous empathy training.

#### Level of empathy

All participants identified a desire to practice in an empathic manner. A few felt empathy levels remained largely unchanged, while many were conscious of burnout. Some participants attributed this to progression through their training, weighing the satisfaction of increased independence with the stress of added responsibility, work hours, and competing priorities. One participant noted “*There’s just so much added responsibility and it increases as you go along in your training, obviously there’s a lot in terms of work hours, and how busy you are, and different projects you’re working on … it goes without saying that quality of life definitely takes a hit, and burnout goes up”*. Some residents noted that empathy levels fluctuated as their quality of life ebbed and flowed. They also acknowledged that residents are less time constrained than staff and anticipated a challenge in maintaining empathy and effective communication under the pressure of independent practice.

#### Other humanities/communications training

Participants described several examples of previous empathy and communications training. These included interviews with standardized patients, traditional didactic lecture, and formal courses in their undergraduate medical training. Participants also noted that role models played a large role in helping them develop empathy and communication skills.

In summary, residents largely felt that their level of empathy declined during medical training and anticipated a further decline upon graduation. Most residents had received some formal empathy training prior, and also described the importance of role models in developing a communication style.

### The comics curriculum facilitated holistic development

Participants were polled on the impact the comics curriculum had on their development as medical practitioners. This included their role as medical learners, medical teachers, and burgeoning physicians. They were asked to discuss the impact the curriculum had on them outside of their medical training.

#### Demonstration of institutional commitment to wellness

Participants voiced an appreciation for the curriculum as medical learners. They saw the curriculum as an institutional commitment to humanism and appreciated the course as a safe environment for them to share and normalize difficult experiences. As one participant noted “*having a forum for that and having a curriculum for that where it shows that the program actually cares about that as well is very important.*”

#### Provided techniques to practice patient-centered approach

Residents were mixed on the impact of the curriculum on their development as physicians. Some felt the curriculum had limited impact on their practice, or that it did not provide skills applicable outside of patients with diabetes. Some felt that while they did not learn any new techniques, they appreciated the course as a refresher of previous knowledge. Others felt the curriculum helped them better understand the perspective of patients with diabetes and highlighted the importance of physician-patient communication. Many felt the curriculum provided techniques to practice a patient-centered approach, especially in challenging situations. Participants also noted that seeing the patient’s perspective helped maintain their empathy when dealing with difficult clinical experiences.

#### Fostered resilience

Participants felt that the group discussions normalized the experience of burnout and helped to build a sense of comradery and resilience amongst the participants.

#### Provided tools to help students tackle challenging situations

One participant stated the course helped them as an educator. They felt it was a reminder of the student perspective, and that it helped them better see the impact of physician-patient interactions on observing medical learners. They also felt they gained techniques to teach students how to tackle challenging situations. They found that the focus on empathy and resilience also served as a reminder for them to check in on the wellbeing of junior learners.

Overall, participants found the curriculum to be impactful. As students, they found it to be a safe space for them to share difficult experiences. As growing physicians, participants appreciated the techniques provided by the course, and found it helpful in illustrating the patient perspective. One participant felt the course served as a reminder of the importance of remaining cognisant of learner well-being. Outside of medical education, participants appreciated the course as a way to normalize burnout and build relationships with their peers.

### Participant appreciated the comics as an educational medium

Participants were asked for their feedback on the content of the comics, their effectiveness as an educational medium, and suggestions for improvement.

#### Positive aspects of comics

Feedback on the comics was largely positive. Participants enjoyed the uniqueness of the medium and believed it made the material more engaging. They felt the comics clearly communicated broad themes of compassion and empathy in a way that facilitated further discussion. As residents in the Adult and Paediatric Endocrinology and Metabolism program, they felt that the scenarios in the comics were practical depictions of issues they might face.

#### As an educational medium

Participants felt the comics were a unique and interactive way to present material. While they almost universally favoured comics over textbooks, they were ambivalent about the relative benefits of comics over mediums like movies or video clips, pointing out individuals have their own preferences. It was also noted that comics presented material in a brief but powerful way, as one participant noted “*it’s a visual message as well, which is a lovely way of doing it … you can say a lot in a picture which could take a chapter of a textbook and get that message across really quickly. And I think that’s the real power of it*”. Participants found the comics to be an effective way to teach foundational concepts, and to act as a springboard for further discussion.

#### Refinement of material

Some participants appreciated the non-skills based focus of the course; however others suggested the comics could be improved by presenting more techniques and frameworks for communication skills. While there was some ambivalence regarding the complexity of the comics, it was suggested that incorporating more complex clinical scenarios could be beneficial for senior learners. As one participant noted “*maybe they should just be refined to a more fellow level. To have more subtleties, like more things we might..see or hear on a day-to-day basis*”. Some suggested topics included situations with more realistic barriers to communication and handling uncomfortable interactions.

In summary, participants largely appreciated the content of the comics. They also appreciated the comics as a unique and educational medium. Feedback on the comics was mixed, with some participants suggesting more complicated subject matter given their level of training, while others appreciating the greater focus on themes of empathy rather than clinical complexity.

### Participant feedback on the curriculum

Participants were polled on the relative importance of each aspect of the curriculum. They were also asked their opinion on the relevance of the curriculum to their overall training. Finally, participants were asked to discuss the logistical implementation of the curriculum.

#### Value of reflective exercises and discussion

Participants were divided on whether the reflective exercises or the group discussion were most beneficial. Most felt the group discussions were an appropriate environment to share experiences and seek advice. Participants largely felt that hearing and sharing experiences was important in promoting self-reflection on challenging situations. One participant felt the reflective exercises played a key role as an intersessional reminder of the topics from previous modules.

#### Relevance of curriculum

Participants were ambivalent on the educational relevance of the curriculum. Some felt that undergraduate medical education sufficiently covered communications training. Others appreciated the course as a reminder of fundamental principles of empathy. Participants described hearing patient complaints of poor physician interactions and noted that such physicians may have benefited from more formalized communications training. One participant appreciated the shift away from a skills-based approach, while others felt the course would be improved by providing more specific techniques to handle challenging scenarios. Participants appreciated the course as their only formalized opportunity for reflection and empathy training. Some felt the focus on diabetes to be overly specific to their program, while others believed the concepts were broadly applicable.

#### Facilitator of curriculum integration

Hosting the session on an academic half-day was preferable as residents felt they could easily disengage from their clinical responsibilities. Participants largely felt the number of sessions were appropriate. They appreciated that the course was interactive and non-didactic, praising its “informal” style. One participant felt the workload outside of the course was appropriate, and that more work could become burdensome.

Overall, feedback on the curriculum was variable. Opinions were mixed on which specific components were most important, though almost all appreciated the opportunity to safely share negative experiences. Opinion on curriculum relevance was also mixed, with some believing they had sufficient exposure from previous training, and others appreciating the course as the sole opportunity for empathy education in their post-graduate education. Participants felt the course to be appropriate in terms of workload and time commitment.

## Discussion

Our study evaluated a comics-based empathy and communication training course for fourth- and fifth-year endocrinology residents. Participants felt the curriculum to be impactful as medical learners, teachers, and practitioners, appreciating the opportunity to share negative experiences and provide support to one another. Participants enjoyed the comics as an educational medium, particularly in their ability to powerfully and concisely communicate the patient perspective. Opinion on the relevancy of the course was mixed, but residents largely appreciated having a course focused on empathy and communications training.

All participants described a desire to practice in an empathic manner. This mirrors our previously reported findings on the importance that undergraduate medical students place on empathy, and suggest these values persist into post-graduate training [11, 12]. Participants acknowledged however, that their levels of empathy tended to fluctuate as they progressed through residency, reporting increased feelings of burnout and impatience. Contributing factors included the stress of increasing responsibility, long work hours, and competing work-life priorities, and are similar to those reported in the literature [8]. The inverse relationship between burnout and empathic practice is well established, and our results highlight the importance of educational interventions at this level to promote wellness.

Numerous studies have described the benefit of empathy training [2, 10, 11, 19, 20], and many novel methods of have been tried, including exposure to poetry and short stories, and having residents role play as patients [21, 22]. However, our paper is the first to investigate the role of a comics-based curriculum in empathy training. A strength of our method lies in the longitudinal nature that an academic curriculum provides. Short term, targeted interventions have been shown to have short term empathy increases, with post-intervention decreases [19, 23]. Repeat, targeted interventions in contrast, showed a sustained improvement in empathy [23].

Participants appreciated the novel use of comics as an educational medium. Depictions of the patient experience helped them better understand the patient perspective, particularly for topics like patient non-compliance, and allowed them to maintain empathy in these situations. Feedback on the comics is in agreement with previously reported benefits [10], with participants reporting that comics were able to powerfully and concisely communicate concepts, while having sufficient depth to inspire further discussion. Residents recalled using depicted techniques when faced with similar situations in a clinical environment, and most participants felt the curriculum had a lasting impact on their empathic practice.

Participants also appreciated the curriculum as an institutional commitment to wellness and humanism. Hearing strategies their colleagues used to navigate challenging situations helped to normalize less successful outcomes. Such feedback is in keeping with a recent study by Daskivich et al. [24] that sought the input of a resident panel to identify interventions to reduce burnout. This panel highlighted the importance of personal support from co-residents and staff, as well as having institutional policies in place to address wellness. While limited to one participant, a notable finding is that the curriculum not only improved the wellness of that participant, but also acted as a reminder of the importance of wellness when taking on junior learners. Such an impact suggests the curriculum may behave as a vehicle for perpetuating resilience in future generations.

Participant feedback on the curriculum was mixed, highlighting the difficulty in designing a curriculum for adult learners. A key principle of adult education is to incorporate the practical experience that students bring [25]. Our participants came from diverse backgrounds with non-standard foundational knowledge. That some felt the curriculum to be redundant, while others appreciated its content is to be expected. Where the course was most successful was in allowing group discussion amongst participants. As senior medical learners, participants possessed a wealth of experience and were themselves resources to their peers. Allowing them to self-direct their education and incorporate their experiences is a key component of adult education, and the positive feedback around this aspect of the course clearly demonstrates its importance [25].

Strengths of this study include the use of a novel intervention to explore empathy and burnout. While we have shown success in the use of comics in junior medical learners, this is the first time we have demonstrated such an impact on the empathic practice and wellness of medical residents. Limitations include a small sample size and a highly specific cohort of participants. Analysis of the data may have been improved by performing member-checking with participants, however the authors did not have the necessary time/resources and have relied on independent coding to ensure methodologic rigour. It may also have been beneficial to perform pre- and post-exposure focus groups/interviews, however the authors felt that conducting only post-exposure data collection would be sufficient for understanding the experience of residents during the curriculum.

## Conclusion

Resident burnout and associated decline in empathy continues to be a pressing issue. The prevalence of burnout in post-graduate medical learners demands novel interventions at an institutional level. Our study used an innovative educational medium in the form of graphic novels to improve resident empathic practice and burnout in a cohort of fourth- and fifth-year endocrinology residents. Participants felt the use of comics to be an effective way to communicate the patient perspective and to demonstrate empathic care, incorporating the techniques shown in the graphic novels into their own practice. They appreciated having dedicated time to wellness in their curriculum and used the opportunity to reflect on challenging situations, and to seek support from their peers and the course facilitator. While challenges remain in optimizing the course for adult learners, the use of graphic novels was universally appreciated. This curriculum serves as a proof-of-concept of the effectiveness of such a medium and could be applied to other medical specialities as well as for junior medical learners.

## Figures and Tables

**Fig. 1:**
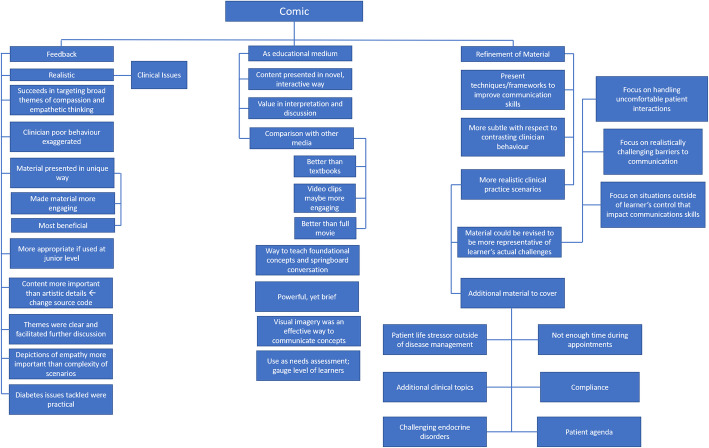
Representative example of framework development. Transcribed data was coded independently by the three authors. Independently generated codes were discussed and refined with subsequent data analysis. After final data analysis, codes were consolidated and representative quotations presented as shown in Table1. As an example, pictured above is an early coding schematic highlighting participant feedback on the comics. The authors examined and discussed these proto-codes to identify relevant and concise themes as elaborated on in Theme 3

## Data Availability

Following research ethics standards, all data is presented in aggregate and individual focus group transcripts are not available. Please contact the corresponding author if you require more details.

## Appendix 1

### Structured Interview Guide

Script and instructions:

Thank you for participating in this study. You have Attended at least one “Empathy, Humanism & Comics” session this academic year. The purpose of this session is to reflect on the curriculum and consider how they affect your interactions with patients.

In this interview, I will be asking you a series of open-ended questions. To help me with gathering and analyzing the results, I will be recording your responses. There won’t be any identifying information on these recordings. Any identifying information will also be removed in the transcription process. They will be kept for 5 years and then the files will be destroyed. The transcribed answers will be secured in a safe place without any identifying information on them.

This focus will last around 30–45 min.

At the end, you will fill out a final survey, which should take less than 5 min.

Do you have any questions before we begin?

Impact of the Curriculum:

The curriculum consisted of 6 sessions over the year (refer to sheet). During each session, you viewed a comic, discussed what was going on, what went well and what could be done better in that comic. Then there was a discussion point around humanism and your role as physician, teacher, person. This was followed by a reflective exercise done in between sessions that was debriefed at the next session.

1. What impact, if any, did this curriculum have on you? (potential cues: How did the curriculum affect your patients throughout the year and in the future? What was its impact on you as a physician? What was its impact on you as a teacher? What was its impact on you as a person? Have you had any previous empathy and communications training? How does this complement your previous empathy and communications training?)
  - If no effect on approach: how could the curriculum be revised to be most useful?
2. Now let’s talk about specific components of the curriculum – comic and debrief, humanism discussion, reflective exercise and debrief. What aspects of the curriculum did you find most valuable and why? What aspects were less useful and why?
  (potential cues: comics themselves, comic debrief, humanism discussion, reflective exercise, reflective exercise debrief; how do you think students can use the comics most effectively – viewing it on their own or discussing it in a group? What are some other reflective activities you’ve done in residency, if any? What is the role, impact, or value of these reflective activities? How was the format of the reflective exercises conducive to the content of the exercises Is there a role of additional reflective exercises?)
3. We are now going to discuss any effect on your approach to patients and empathy:
  - How do you feel your level of empathy has changed since you started your medical training?
  - What were the key issues that stood out for you when you reflect back on the curriculum? What was your awareness of the issue before and how did it change?
    i. What was your attitude towards patient with diabetes? How did it change?
    ii. What was your knowledge of the patient’s perspective (views, feelings, fears) on living with diabetes? How did it change?
    iii. What differences exist between patient and physician perspectives? How did your knowledge of this difference change?
  - What is the impact of this curriculum on your level of empathy or your empathic approach to patients?
    i. If no impact: how could the curriculum be revised to be most useful?
4. We are now going to discuss the potential educational value of this curriculum, and comics, if any.
  - What have you learned so far in medical residency about chronic disease and communication skills?
  - How does this curriculum compare or add to what you’ve learned so far?
  - What do you think of comics as a medium for learning?
  - How do comics compare as a learning medium to other formats, like text, movies or video clips?
  - How do you think the comics can be best integrated into the current curriculum?
  - How do you think students can use the comics most effectively – viewing it on their own or discussing it in a group?

What other suggestions do you have for improving the curriculum and comics?

5. Any other final comments?
